# Supplementary material for: Evaluation of Solar-Driven Photocatalytic Activity of Thermal Treated TiO2 under Various Atmospheres
Source: Nanomaterials (Basel). 2019 Jan 29;9(2):163. doi: 10.3390/nano9020163 (PMC6409930; doi:10.3390/nano9020163)
Supplement: Supplementary file 1 [file nanomaterials-09-00163-s001.pdf]

Supporting Information

Evaluation of Solar-Driven Photocatalytic Activity of Thermal Treated TiO_2 under Various Atmospheres

Reza Katal [1], Saeideh Kholghi Eshkalak [2], Saeid Masudy-panah [3], Mohammadreza Kosari [4], Mohsen Saeedikhani [5], Mehrdad Zarinejad [6,*] and Seeram Ramakrishna [2,*]

1 Department of Civil & Environmental Engineering, National University of Singapore, Singapore 117576, Singapore; reza.katal@u.nus.edu
2 Department of Mechanical Engineering, Center for Nanofibers and Nanotechnology, National University of Singapore, Singapore 117575, Singapore; s.kholghi143@gmail.com
3 Department of Electrical and Computer Engineering, National University of Singapore, Singapore 119260, Singapore; saeid1@e.ntu.edu.sg
4 Department of Chemical and Biomolecular Engineering, Faculty of Engineering, National University of Singapore 119260, Singapore; kosari@u.nus.edu
5 Department of Materials Science and Engineering, National University of Singapore, Singapore 117583, Singapore; saeedikhani@u.nus.edu
6 Singapore Institute of Manufacturing Technology (SIMTech), A*STAR (Agency for Science, Technology and Research), Singapore 138634, Singapore
* Correspondence: mehrdad@simtech.a-star.edu.sg (M.Z.); seeram@nus.edu.sg (S.R.); Tel.: +65-6516-4805 (S.R)

Table S1. The surface area of samples.

Samples	Surface area (m^2/g)
P25	50.1
A-400	38.2
V-400	37.9
H-400	37.8

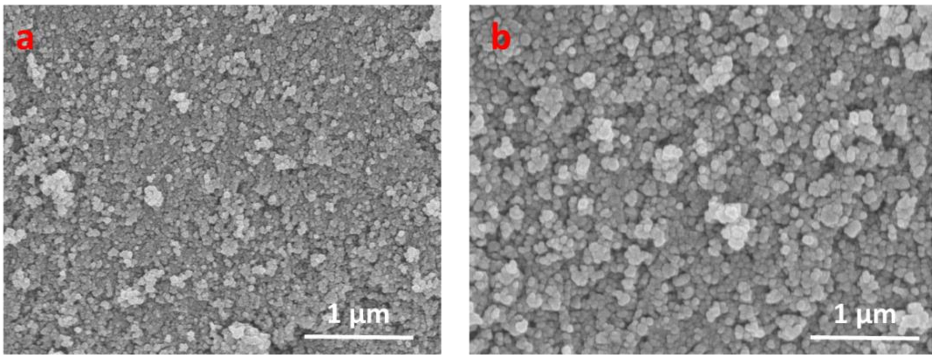

Figure S1. The SEM images of (**a**) P25 and (**b**) H-400.

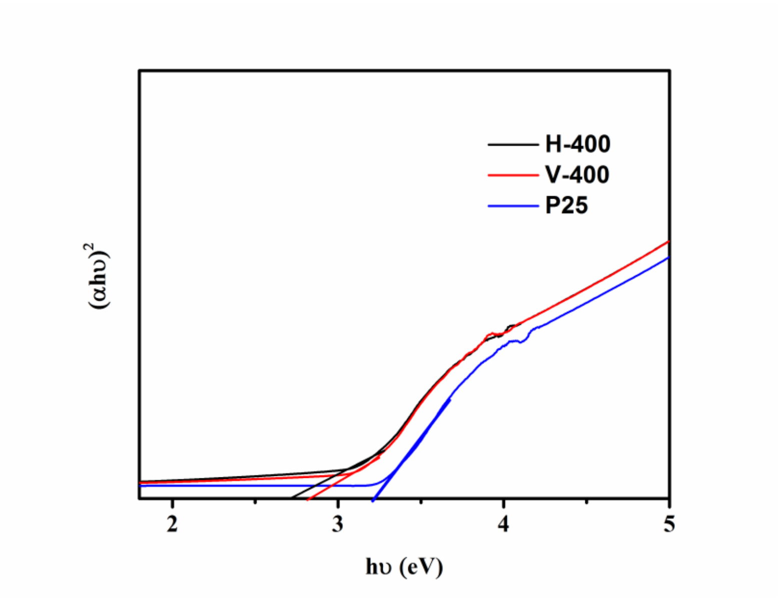

Figure S2. Plot of $(\alpha h\upsilon)^2$ versus hυ for H-400, V-400 and P25.

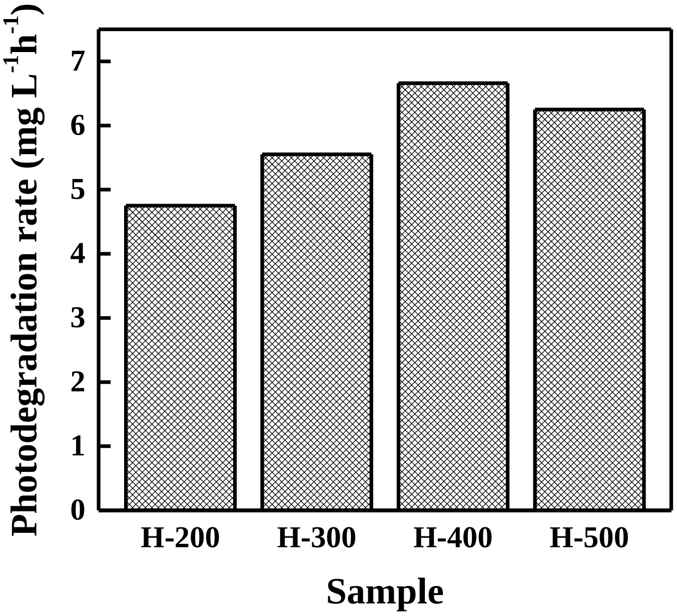

Figure S3. The photocatalytic degradation rate of hydrogenated samples under 1.5 G solar light illumination.

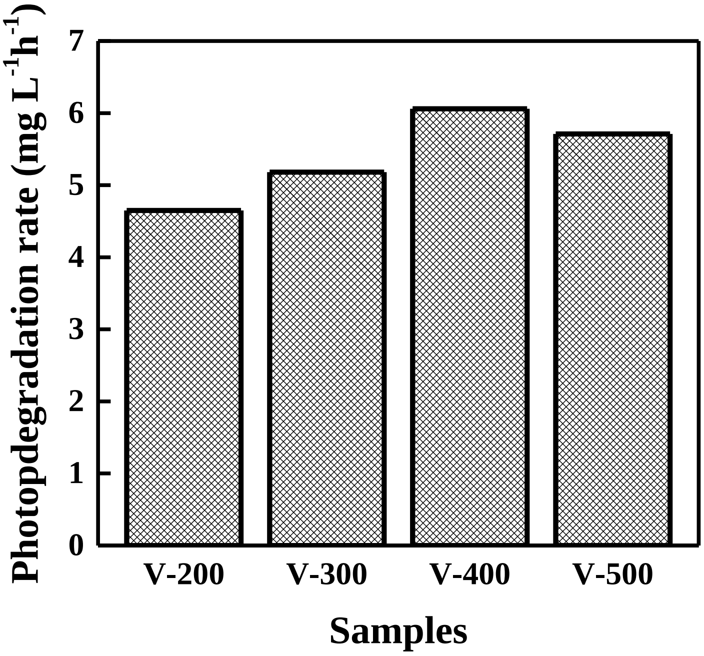

Figure S4. The photocatalytic degradation rate of calcined samples under vacuum under 1.5 G solar light illumination.

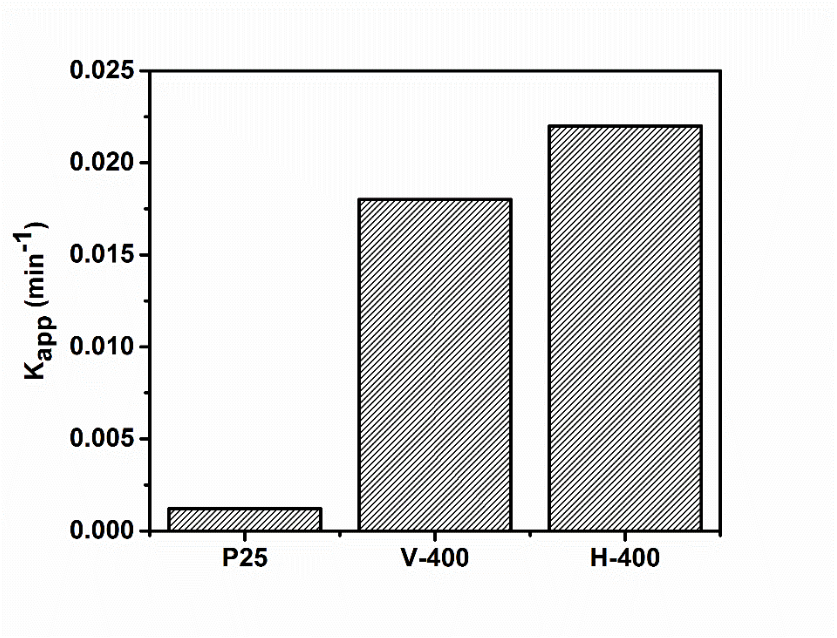

Figure S5. K_{app} (min^{-1}) of the samples under visible light irradiation.

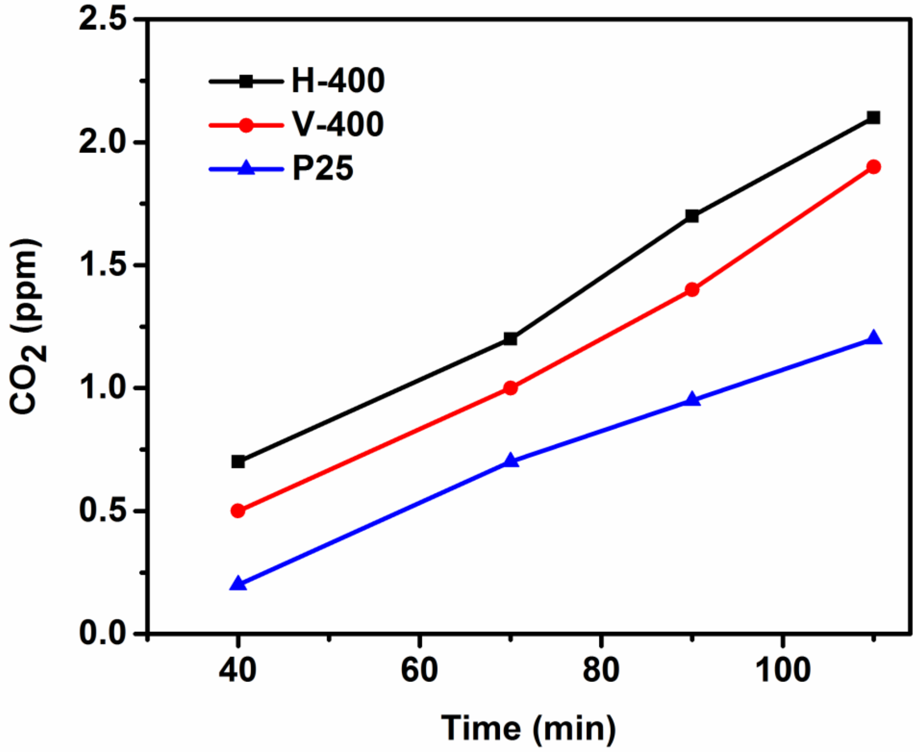

Figure S6. CO_2 production during the photocatalytic degradation of MB.

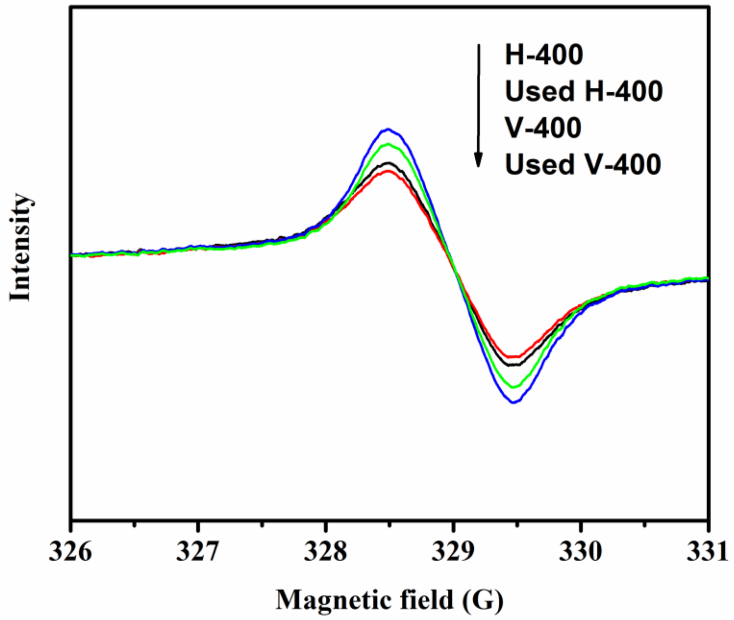

Figure S7. EPR spectra recorded at room temperature for fresh and used samples.

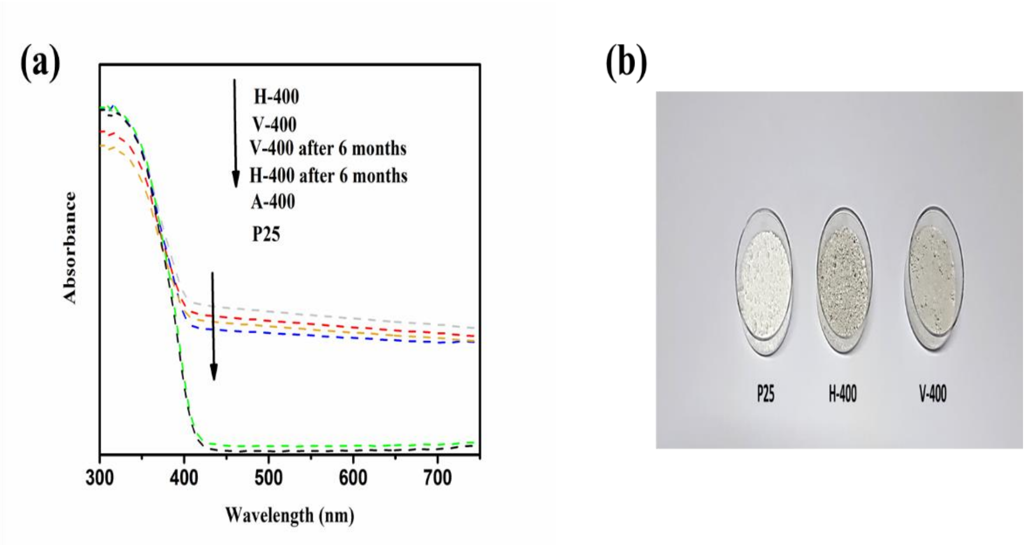

Figure S8. (**a**) UV-vis spectra and (**b**) images of samples after 6 months storage.
